# Does the use of store-and-forward telehealth systems improve outcomes for clinicians managing diabetic foot ulcers? A pilot study

**DOI:** 10.1186/1757-1146-4-S1-P31

**Published:** 2011-05-20

**Authors:** Peter A Lazzarini, Damien Clark, Rebecca D Mann, Vanessa L Perry, Courtney J Thomas, Suzanne S Kuys

**Affiliations:** 1 Allied Health Research Collaborative, Metro North Health Service District, Queensland Health, Brisbane, Australia; 2 Department of Podiatry, Metro North Health Service District, Queensland Health, Brisbane, Australia; 3 School of Public Health, Queensland University of Technology, Brisbane, Australia; 4 Department of Podiatry, Metro South Health Service District, Queensland Health, Brisbane, Australia; 5 Clinical and Statewide Services Division, Queensland Health, Brisbane, Australia; 6 Department of Allied Health, Mount Isa Health Service District, Queensland Health, Mount Isa, Australia; 7 School of Physiotherapy and Exercise Science, Griffith University, Brisbane, Australia

## Background

Diabetic foot ulcers are one of the most hospitalised diabetes complications and contribute too many leg amputations. Trained diabetic foot teams and specialists managing diabetic foot ulcers have demonstrated reductions in amputations and hospitalisation by up to 90%. Few such teams exist in Australia. Thus, access is limited for all geographical populations and may somewhat explain the high rates of hospitalisation. This pilot study aims to analyse if local clinicians managing diabetic foot complications report improved access to diabetic foot specialists and outcomes with the introduction of a telehealth store-and-forward system.

## Method

A store-and-forward telehealth system was implemented in six different Queensland locations between August 2009 and February 2010. Sites were offered ad hoc and/or fortnightly telehealth access to a diabetic foot speciality service. A survey was sent 6 months following commencement of the trial to the 14 eligible clinicians involved in the trial to gauge clinical perception of the telehealth system.

## Results

Eight participants returned the surveys. The majority of responding clinicians reported that the telehealth system was easy to use (100%), improved their access to diabetic foot speciality services (75%), improved upskilling of local diabetes service staff (100%), and improved patient outcomes (100%).

## Conclusion

This pilot study suggests that clinician’s found the use of a telehealth store-and-forward system very useful in improving access to speciality services, clinical skills and patient outcomes. This study supports the recommendation that telehealth systems should be made available for diabetic foot ulcer management.

